# Ethnobotany, Cytotoxicity and Color Stability of Karen Natural Colorants

**DOI:** 10.3390/plants14091348

**Published:** 2025-04-29

**Authors:** Sukhumaabhorn Kaewsangsai, Prateep Panyadee, Aussara Panya, Hataichanok Pandith, Prasit Wangpakapattanawong, Henrik Balslev, Angkhana Inta

**Affiliations:** 1 Department of Biology, Faculty of Science, Chiang Mai University, Chiang Mai 50200, Thailand; sukhumaabhorn@gmail.com (S.K.); aussara.pan@cmu.ac.th (A.P.); hataichanok.p@cmu.ac.th (H.P.); prasit.wang@cmu.ac.th (P.W.); 2 Queen Sirikit Botanic Garden, The Botanical Garden Organization, Chiang Mai 50180, Thailand; pt.panyadee@gmail.com; 3 Department of Biology, Faculty of Natural Science, Aarhus University, DK-8000 Aarhus, Denmark; henrik.balslev@bio.au.dk

**Keywords:** ethnobotany, local plants, natural dye, plant-based dye, traditional knowledge

## Abstract

Historically, natural pigments have been used to color textiles, food, and cosmetics, but the rise of synthetic dyes in the 19th century reduced their use. Recently, interest in plant-based pigments has surged due to health and environmental concerns. Among Thailand’s ethnic groups, the Karen use plant colorants extensively, but their practices remain understudied. In this study, we document the knowledge of plant colorants of the Karen community in Chiang Mai province, assess the color stability of the plant colorants, and evaluate their cytotoxicity in HepG2 cells. Interviews with 113 informants revealed 52 plant species used for dyeing, primarily through hot water extraction. The most common color was brown, and *Strobilanthes cusia* had the highest color use value (0.93). The study identified 10 color shades, with blue showing the highest consensus. Color stability was tested at room and elevated temperatures, which showed that colorants from *Oroxylum indicum* were the most stable, and those of *Strobilanthes cusia* had the lowest toxicity (CC_50_ = 994.1 µg/mL), while *Artocarpus lacucha* had the highest toxicity (CC_50_ = 63.96 µg/mL). *Oroxylum indicum*, which has excellent color stability and moderate cytotoxicity (CC_50_ = 294.4 µg/mL), is recommended as a promising natural colorant. This study provides valuable insights into preserving traditional knowledge in Karen communities.

## 1. Introduction

Throughout history, people across the world have developed methods for extracting and using natural pigments to color textiles, food, and cosmetics [1,2]. The discovery of synthetic colorants in the 19th century marked a turning point in the use of natural dyes. British chemist William Henry Perkin produced the first synthetic organic pigment, mauveine, from aniline [3]. Following this breakthrough, synthetic colorants gained widespread popularity due to their lower cost, greater availability, and enhanced stability [4]. Consequently, the use of natural colorants and the traditional knowledge associated with them have declined as they have been replaced by synthetic alternatives.

However, in recent years, the demand for natural pigments, particularly those derived from plants, has surged due to their eco-friendly properties and minimal toxicity [5]. Advances in plant-based dye technology have facilitated their application in textiles, food, cosmetics, and pharmaceuticals [6]. The food industry has shown growing interest in plant-derived colorants as alternatives to synthetic dyes, driven by consumer demand for natural products [7]. Regulatory bodies such as the World Health Organization (WHO) emphasize the need for safety evaluations of food colorants before their approval for public use [8]. Cytotoxicity testing is a widely used method in this context [9]. However, replacing synthetic dyes remains challenging because natural pigments are often susceptible to degradation by heat, light, and oxygen exposure [10,11]. The stability of plant-based pigments varies among species, necessitating further investigation into their color retention properties [12,13].

Increasing awareness of health and environmental sustainability has contributed to renewed interest in natural plant-based colorants [14]. Consumers seek products that promote well-being while minimizing damage to the environment [15]. As a result, extensive research is being conducted to identify new plant pigment sources as viable alternatives to synthetic colorants.

The ethnobotanical knowledge of natural dyes has been preserved by various ethnic communities worldwide. For instance, the Mazahua people in central Mexico use 29 plant species for dyeing textiles to preserve their cultural heritage [16]. Similarly, 29 species have been documented in traditional Italian dyeing practices [17]. Ethnobotanical studies have documented traditional dyeing practices in China, Vietnam, Iraq, and Turkey, highlighting the continued use of plant-based colorants in these regions [4,18,19,20].

In Thailand, plant-derived colorants remain an integral part of the traditions of some ethnic groups’. Previous studies have documented 212 plant species used for dyeing purposes throughout the country [21,22,23,24,25,26]. Among these, the Karen ethnic group has been identified as a primary custodian of traditional dyeing practices [21,22,25,27,28,29,30]. The Karen extensively use natural dyes for their handwoven textiles, emphasizing the cultural significance and uniqueness of their craftsmanship [18,19].

The Karen are the largest ethnic group in Thailand, predominantly residing in the Chiang Mai, Tak, and Mae Hong Son provinces [31]. Originally from Myanmar, they migrated to Thailand in the 18th century, and today, the Thai Karen population is estimated to be 550,000 [32]. The Karen are traditionally classified into four subgroups: Skaw, Pwo, Bwe, and Taungthu. They typically inhabit areas at elevations of more than 500 m above sea level and rely on swidden agriculture and livestock farming for subsistence [33].

Renowned for their weaving skills, the Karen produce distinctive handwoven textiles that are dyed with natural pigments. Their traditional attire varies according to age and marital status. Young girls wear long white cotton dresses until marriage, while married women don skirts with blue, white, or black stripes paired with v-necked sleeveless blouses in dark colors. Men wear sleeveless cotton shirts featuring red, white, or yellow vertical stripes, distinguished by a deep v-neck [33,34].

Many plants used as colorants among the Karen also have edible or medicinal properties, suggesting their potential applications in food, cosmetics, and pharmaceuticals [20,35]. Despite the ethnobotanical documentation of these dye plants, comprehensive studies on their chemical properties, cytotoxicity, and color stability are scarce.

This study aims to investigate the ethnobotanical knowledge, cytotoxicity, and color stability of plant-based colorants used by the Karen community in Chiang Mai province. Specifically, it seeks to answer the following questions: How many and which plant species are used as colorants among the Karen communities in Chiang Mai province? Which plant-derived colorants are the most significant in Karen culture? What are the cytotoxicity levels and color stability properties of these plant colorants? By addressing these questions, this research intends to contribute to the preservation of traditional knowledge while assessing the viability of plant-based pigments as sustainable alternatives to synthetic dyes.

## 2. Results

### 2.1. Natural Colorants Used by Karen Communities in Chiang Mai Province

We gathered 1398 use reports from six Karen villages covering six districts in Chiang Mai province. The use report related to 52 plant colorant species belonging to 49 genera and 30 families (Table 1). Fabaceae had the highest number of plant colorant species (16 species, 142 use reports), followed by Anacardiaceae (4 species, 150 use reports), Lamiaceae (3 species, 114 use reports), Pentaphylacaceae (3 species, 95 use reports), Moraceae (3 species, 94 use reports) and Fagaceae (3 species, 63 use reports). We found that 67% (20 families) were represented by one species with plant colorant use.

The number of species used was 26 in Khun Tuen Noi, 18 in Khuntae, 21 in Mae Hae Tai, 37 in Huay E Khang, 26 in Wat Chan, and 15 in Pakanok.

Plant colorant species may have different life forms. The most common life forms of the plant colorant species were trees (26 species), followed by shrubs (14 species), herbs (9 species), scandent (2 species), and palms (1 species).

The most commonly used plant part for dyeing was the bark. It was mentioned in 593 use reports, followed by fruits (317 use reports), leaves (210 use reports), rhizomes (94 use reports), stems (78 use reports), roots (70 use reports), seeds (41 use reports), flowers (33 use reports) and whole plants (six use reports).

### 2.2. Important Plant Colorant Species and Color Shade of Karen

**Figure 1:**
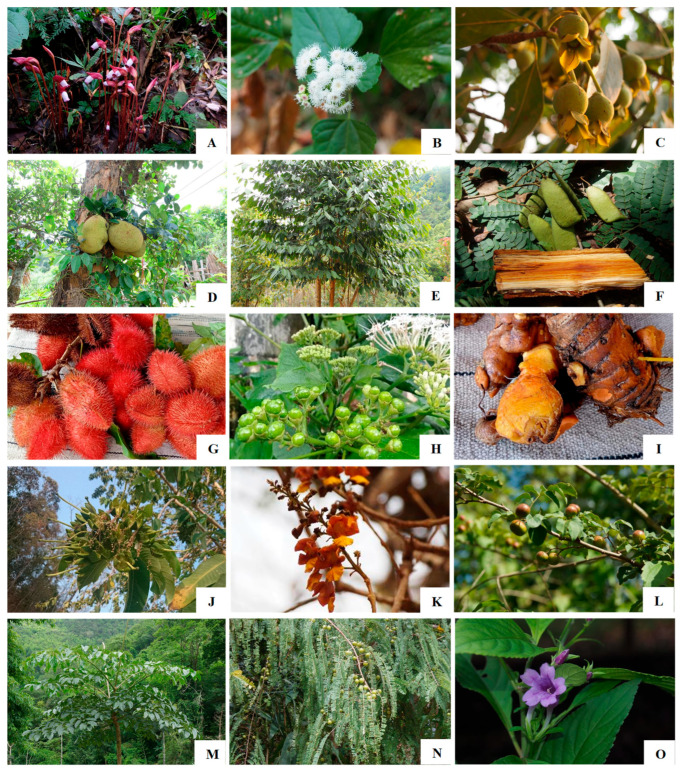
Some important plant colorant species used by the Karen in Chiang Mai Province, Northern Thailand including (**A**) *Aeginetia indica*, (**B**) *Ageratina adenophora*, (**C**) *Anneslea fragrans*, (**D**) *Artocarpus heterophyllus*, (**E**) *Artocarpus lacucha*, (**F**) *Biancaea sappan*, (**G**) *Bixa orellana*, (**H**) *Clerodendrum glandulosum*, (**I**) *Curcuma longa*, (**J**) *Duabanga grandiflora*, (**K**) *Gmelina arborea*, (**L**) *Harrisonia perforata*, (**M**) *Oroxylum indicum*, (**N**) *Phyllanthus emblica*, and (**O**) *Strobilanthes cusia*.

The use value (UV) of plant colorant species employed by various ethnic groups in northern Thailand ranged from 0.01 to 0.93, with an average of 0.23 (Table 1). A total of 20 species had use values (UV) exceeding the average, indicating their significant role in traditional dyeing practices (Figure 1). Among these, *Strobilanthes cusia* (UV = 0.93), *Clerodendrum glandulosum* (UV = 0.86), and *Curcuma longa* (UV = 0.82) were the most frequently used species. In contrast, the lowest UV values (0.01) were recorded for *Adinandra integerrima*, *Castanopsis acuminatissima*, *Diospyros mollis*, *Dipterocarpus obtusifolius*, *Jatropha gossypifolia*, *Pterocarpus macrocarpus*, and *Terminalia phillyreifolia*, each of which was mentioned only once by the informants.

The fidelity level (FL) was calculated to determine the predominant color shade extracted from each plant. Among the 13 species with the highest FL (100%), five were used for brown, two for green, two for red, and one each for blue, gray, pink, and purple colors. Biancaea sappan exhibited the most diverse range of color shades, with pink being the most prominent (FL = 85%), followed by red (5%), yellow (3%), orange (3%), purple (2%), and brown (2%) (Table 2).

The homogeneity of the plant colorant species used for each color shade was assessed using the informant consensus factor (ICF), which ranged from 0.20 to 0.97 (Table 3). Blue had the highest ICF value (0.97) based on 149 use reports and six dye plant species, followed by gray (ICF = 0.94, 229 use reports, 14 species) and green (ICF = 0.93, 195 use reports, 10 species).

### 2.3. Traditional Dyeing Process of Karen

The traditional knowledge techniques of natural dyeing of the Karen have been passed down through the generations. The wisdom of previous generations and knowledge of natural dyeing have been used to color fabrics using non-toxic materials derived from nature. This cultural heritage is passed on to descendants and serves as a livelihood tool for rural communities.

#### 2.3.1. The Fabric or Yarn Preparation

**Figure 2:**
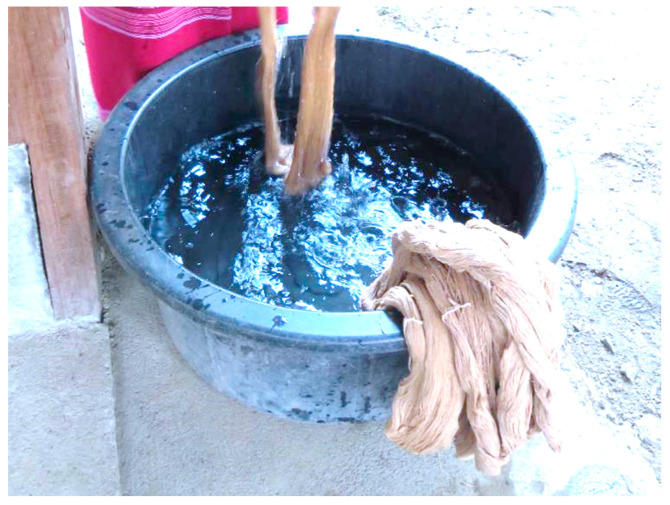
Before dyeing, the yarn undergoes a preparation process to ensure optimal color absorption and dye adherence.

The fabric or yarn was completely soaked in clean water prior to dyeing. The cotton fibers were then boiled for 30 min in water that had been treated with a synthetic detergent. Boiling the yarn eliminated dirt, fat, protein, and any remaining starch that had adhered to its surface. The cotton fiber was left to drip off the extra water, or the water was pressed off (Figure 2).

#### 2.3.2. Mode of Preparation and Dye Extraction

**Figure 3:**
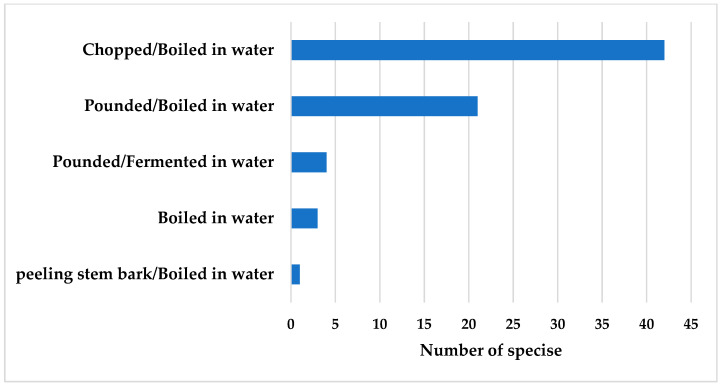
Number of plant colorant species used in each preparation method.

Most of the preparation and dye extraction of plant colorants used by 113 informants and 1398 use reports depended on the available part of the plant materials (Figure 3). The raw materials were cut into small pieces, and plant materials were obtained through boiling, soaking, or fermenting with water. The dye extraction process was performed using the following two methods:

- Hot extraction method

**Figure 4:**
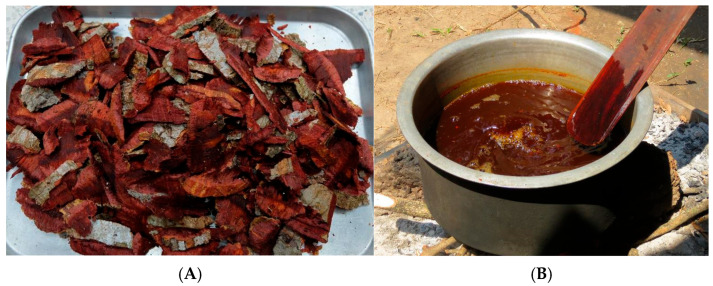
Plant material preparation and dye extraction. (**A**) bark of *Anneslea fragrans* was cut into small pieces before the extraction process; (**B**) dye solution obtained from *Quercus brandisiana* after the extraction process.

The plant materials were boiled in water. Heat acts as a catalyst to bring out the pigments in the plant. The plant colorant species used in the hot extraction method included *Anneslea fragrans*, *Duabanga grandiflora*, and *Quercus brandisiana*. The mixed solution was then filtered with cotton fabric to obtain the dye solution (Figure 4).

- Cold extraction method

**Figure 5:**
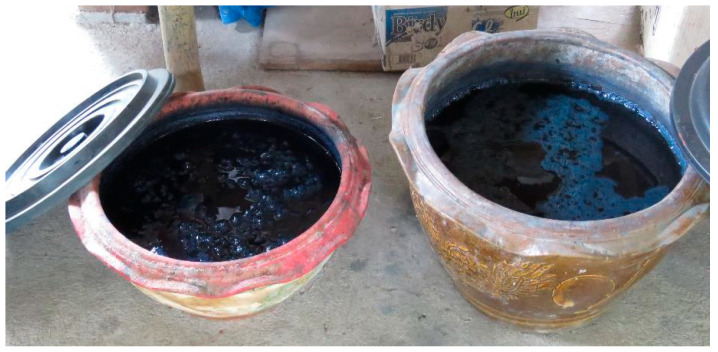
The dye solution was obtained from *Indigofera tinctoria* after the extraction process.

Some plant colorant species, such as *Indigofera tinctoria* and *Strobilanthes cusia* were extracted by the cold method. Fresh leaves and stems were collected from the plants. The fermentation process was completed when the stems and leaves became mushy and black. To ensure that all the leaves were soaked and fermented, the extract solution was stirred up and down once a day with a wooden rake every two to three days to add air to the solution. This was repeated for one month to obtain a ready-to-use dye solution (Figure 5).

#### 2.3.3. Dyeing Methods

**Figure 6:**
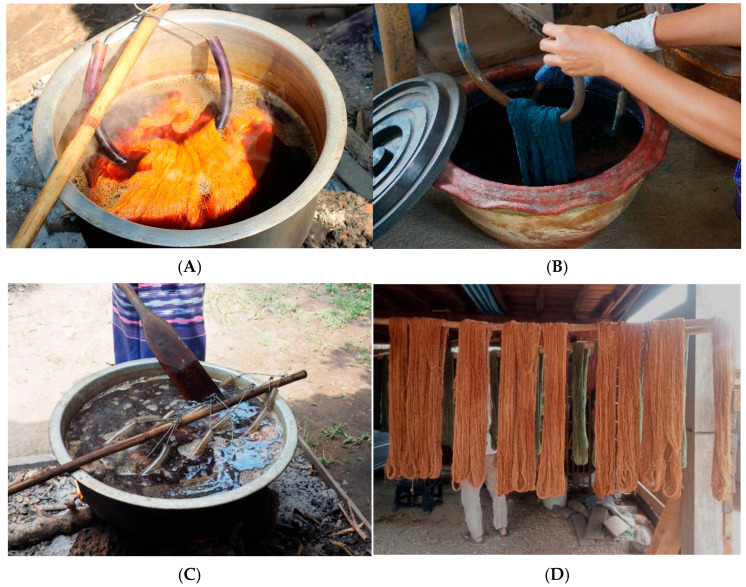
The dyeing process of yarn (**A**,**B**) The yarns were immersed in the prepared dye extraction solution; (**C**) Utilize a spatula to press the yarn into the dye solution; (**D**) The dyed yarns were suspended on strings in the shade to naturally dry.

Following the fabric or yarn preparation and dye extraction process, the yarns were placed in the prepared dye solution. A spatula was used to ensure that the cloth was fully submerged, pressing out any air bubbles. The fabric was occasionally stirred with a spatula to ensure even coverage. The samples were soaked for 30–60 min to allow the dye to adhere. After the designated time, excess dye was pressed from the yarn, which was then left to dry. The yarn was submerged three to four times until the desired color was achieved. Alternatively, the fabric can be left in the dye bath until the preferred shade is achieved, and the dye bath can then be preserved for reuse. Consequently, the color intensity increased with each dyeing cycle. Finally, the dyed fabric was removed from the mixture and washed with cold water. It was rinsed until the water ran clear before being hung on strings to air dry in the shade (Figure 6).

### 2.4. Additional Usages of the Plant Colorants

The Karen people use plant colorant species to dye a variety of items, including food, medicine, and materials, and for ceremonial purposes (Table 4). For instance, *Mangifera indica*, *Artocarpus heterophyllus*, *Artocarpus lacucha*, and *Morus alba* have edible fruit varieties that are consumed as food. Some plant colorant species were cooked and served with chili paste, such as young fruits of *Oroxylum indicum* and young shoots of *Clerodendrum glandulosum*, which were boiled as vegetables.

Because of their medicinal properties, some plant-colorant species are used by the Karen to maintain their health. The *Biancaea sappan*’s stem was decocted and used as a blood tonic and nourishing remedy for the body. The raw fruit of *Phyllanthus emblica* is consumed to treat sore throats.

In terms of materials and other applications, the Karen people utilize the leaves of *Livistona speciosa* to build roofs, and the leaves of *Anneslea fragrans* are chewed with betel nut, a practice also observed with the bark of *Artocarpus lacucha*.

### 2.5. Color Stability and Cytotoxicity of Plant-Based Colorants Used by the Karen

Fifteen plant colorant species with high ethnobotanical indices (UV ≥ 0.33) were selected based on their wide distribution, accessibility, and natural abundance. These species were also cultivated in the Karen home gardens for use. The color stabilities of the 15 heat-treated solid dye powders were assessed using the color difference parameter ΔE* (Table 5). The highest color change was observed in *Phyllanthus emblica* stored at room temperature (ΔE* = 8.86), followed by *Strobilanthes cusia* at room temperature and *Phyllanthus emblica* at 45 °C. In contrast, *Oroxylum indicum* exhibited the lowest color change when stored at 45 °C (ΔE* = 4.64), followed by *Oroxylum indicum* at room temperature and *Curcuma longa* at 45 °C.

The cytotoxic activities of fifteen plant colorant extracts were evaluated against the HepG2 cell line. The extracts were tested at concentrations ranging from 1.6–1000 μg/mL, and cell viability was assessed after 48 h using the PrestoBlue cell viability assay. Cytotoxicity was assessed based on CC_50_ values, with concentrations maintaining cell viability above 80% classified as non-toxic in accordance with the ISO 10993-5 guidelines. To further illustrate the non-toxic dose range, a bar graph is provided as Supplementary Data (Supplementary Figure S1). The cytotoxicity results, summarized in Table 6 and derived from three independent experiments, with *Cordyceps militaris* extract used as a reference in this study, showed that *Strobilanthes cusia* had the highest CC_50_ value (994.1 µg/mL), indicating the lowest cytotoxicity, while *Artocarpus lacucha* had the lowest CC_50_ value (63.96 µg/mL), suggesting the highest cytotoxicity. *Strobilanthes cusia* primarily contains indole alkaloids as color-related active compounds, including indigo, indirubin, and isatin. *Artocarpus lacucha* contains oxyresveratrol, which exhibits skin-whitening and anti-aging properties.

## 3. Discussion

### 3.1. Colorant Species Among the Karen Communities in Chiang Mai Province

The Karen ethnic communities in Chiang Mai use a highly diverse range of plant-based colorants. Based on comparisons with previous studies, these plants account for 27% of all known plant colorant species in Thailand. This finding highlights the significant prevalence of plant colorant use among the Karen ethnic groups in this region [23,24].

Among these plant colorants, Fabaceae is the most dominant, which is consistent with ethnobotanical research conducted across various ethnic groups in Thailand. Fabaceae has consistently been one of the most mentioned plant families in ethnobotanical surveys, with the highest number of reported uses and species [23,24,36,37,38,39]. Additionally, Fabaceae has been reported as the leading source of plant colorants among ethnic communities in northern India, Vietnam, and Myanmar [20,40].

Globally, Fabaceae is the third-largest plant family, comprising over 700 genera and approximately 19,500 species, ranking behind Asteraceae and Orchidaceae in species richness. It is widely distributed across all biomes. Due to its large size and diverse life forms, Fabaceae is the most commonly used plant family for natural dyes among ethnic groups in northern Thailand. Moreover, it has been identified as having the highest family use value for medicinal plants both in Thailand and worldwide [41,42,43,44].

Previous research on Karen plant colorant species in northern Thailand has documented the use of over 60 species. This study identified eight additional plant colorant species used by the Karen. For instance, *Duabanga grandiflora*, traditionally used for construction by the Karen [25], and *Leucaena leucocephala*, commonly consumed as a vegetable [45], were both successfully extracted as dye plants, as recorded in the present study.

Our study identified ten distinct color groups derived from nine different plant parts, highlighting the diversity of plant-based dyes used by the Karen. Various plant parts have been used for dye extraction, demonstrating a wide range of sources for obtaining natural colors. A total of 52 species contributed to the ten different color shades. Among these, brown, red, and purple were the most commonly produced colors, each originating from 17 to 23 plant species. The middle group included yellow, green, gray, and orange, which were extracted from 13 to 14 species. The final group, consisting of pink, blue, and black, had fewer sources, with only four to eight plant species per color. The diversity of plant-based dyes has been widely reported in previous studies. This investigation identified ten color hues consistent with findings from northern Thailand [23,46]. In contrast, the Chin ethnic community in western Myanmar was reported to obtain six different colors from plant dyes [40], which is comparable to the findings from northwestern Argentina [47].

Many of the plant colorant species reported here are also used for textile dyeing in different countries. For example, we found that *Biancaea sappan* (heartwood) produced a pink-red dye, whereas it has been reported to yield a brown-purple color in China. Similarly, indigo-blue dye derived from *Strobilanthes cusia* is widely used in textile dyeing in multiple cultures. This species is commonly used by various ethnic groups in Thailand [48,49,50], China [51,52,53], and Myanmar [40].

In some cases, different plant species can yield the same colors. For instance, the black dye can be obtained from the fruit of *Diospyros mollis* and *Harrisonia perforata*, while orange dye can be extracted from the seeds of *Bixa orellana*, as well as from the bark of *Buchanania cochinchinensis* and *Anneslea fragrans*, and the rhizomes of *Curcuma longa*.

Bark was the most frequently used plant part for dye extraction, particularly for producing brown shades. This aligns with previous research on plant dyes used by the Tai Lao ethnic group in northeastern Thailand, which reported brown as the predominant color [23]. The prevalence of brown, yellow, gray, and black dyes derived from plant sources is often linked to the presence of tannins, which are primarily found in the bark [54]. Tannins contribute to a variety of color shades in dyeing processes, a finding consistent with studies on plant-based dyes in northeastern Thailand, northeast India, and Sierra Leone [23,55,56]. The widespread presence of tannins in numerous plant species underscores their importance as natural dye sources, which have been used for centuries in traditional textile coloring.

### 3.2. The Most Important Plant-Derived Colorants in Karen Culture

A high use value indicates the most preferred plant species for dyeing. Among the Karen, *Strobilanthes cusia* is the most significant plant colorant species. It was reported in every village surveyed in this study and had the highest use value (0.93) based on 107 use reports. This aligns with the high ICF index values recorded for a blue dye obtained from *S. cusia*. A high ICF value suggests greater cultural significance and widespread use, indicating that this species plays a crucial role in traditional dyeing practices. The strong agreement among informants underscores the well-established heritage of plant colorant use [57].

*Strobilanthes cusia* is a perennial herb native to South Asia and the Indo-Chinese Peninsula. It propagates easily through cuttings and can be harvested 2–3 times a year for its leaves [58,59]. Several ethnobotanical studies across Asia have documented its use as a plant colorant in India [60,61,62], China [19,51,52,53], and Myanmar [40]. Renowned for yielding indigo, *S. cusia* remains one of the primary sources of natural blue dye across East and Southeast Asia [53].

The homogeneity of plant colorant species within each color shade was assessed using the information consensus factor (ICF), which ranged from 0.20 to 0.97. The blue shade exhibited the highest ICF value, as it was derived from relatively few species, primarily *S. cusia* and *Clerodendrum glandulosum*, both of which had high-use values. In contrast, the brown shade, despite having many use reports, was obtained from a larger number of plant colorant species, leading to a lower ICF value. A high ICF indicates a strong consensus among informants regarding the traditional knowledge of plant colorants, reflecting the cultural importance of specific dye sources [62,63].

The blue color group exhibited the highest Informant Consensus Factor (ICF) value, with plants producing blue dyes also having high Use Value (UV) scores. Most plants producing blue dye are either annuals or perennial herbs, making them suitable for cultivation and harvesting multiple times each year. Notably, *Strobilanthes cusia* and *Clerodendrum glandulosum* are widely used due to their adaptability and diverse applications.

In contrast, the black color group had the lowest ICF value, with each plant in this category receiving only one to two use reports. Species such as *Harrisonia perforata* and *Diospyros mollis* are primarily wild plants found at elevations of 500–800 m above sea level. However, the villages surveyed in this study are located at higher elevations, between 800 and 1400 m, where these species are less common. Compared to blue dye plants, which are widely cultivated, black dye plants are less frequently used, resulting in lower familiarity among the Karen people. This aligns with ethnobotanical studies from the Republic of Georgia in the Caucasus, which found that cultivated plants tend to have more documented uses than wild plants, as reflected in their higher UV values [64].

Although red and black dyes are significant elements in traditional Karen attire [33], the plant species in these dye groups had relatively low ICF values. In contemporary practice, the Karen people primarily wear their traditional clothing on special occasions. Natural-dyed fabrics are now more commonly produced for commercial purposes, serving as a source of household income rather than for personal use. The labor-intensive and complex nature of the natural dyeing process has led many Karen weavers to prefer pre-dyed cotton for clothing production. This trend is consistent with a previous study on Karen textile traditions [65].

### 3.3. The Color Stability Properties and Cytotoxicity Levels of Karen Plant Colorants

This study observed that the lightness (L*), Red-Green Axis (a*), and Yellow-Blue Axis (b*) values of all samples increased when 15 dye powders were stored at different temperatures for 0, 15, and 30 days under both storage conditions. The increase in the L*, a*, and b* values indicates that the dye powders became progressively lighter, redder, and more yellow over time. These color shifts may result from oxidation, chemical reactions, or environmental factors affecting the dye powders during storage [66,67,68].

ΔE* is a key metric for quantifying color differences, representing the perceived distance between two colors in a color space. It is widely used in industries such as printing, manufacturing, and design to assess and maintain the consistency of color [69]. A higher ΔE* value indicates a greater color difference, with a value of 0 signifying identical colors while increasing values denote greater variation [70].

The ΔE* values of *Anneslea fragrans*, *Artocarpus lacucha*, *Quercus brandisiana*, *Syzygium cumini*, and *Terminalia chebula* stored at 45 °C were higher than those of the control samples. In contrast, the ΔE* values of *Biancaea sappan*, *Bixa orellana*, *Buchanania cochinchinensis*, *Curcuma longa*, *Duabanga grandiflora*, *Mangifera indica*, *Oroxylum indicum*, *Phyllanthus emblica*, *Salix tetrasperma*, and *Strobilanthes cusia* were lower than the control samples when stored at 45 °C. However, no statistically significant differences (*p* > 0.05) were observed between the samples stored at room temperature and those stored at 45 °C. This finding is consistent with previous research on the effect of temperature on blueberry drying, which found that drying at temperatures below 50 °C resulted in minimal color changes for both highbush (*Vaccinium corymbosum*) and wild (*Vaccinium angustifolium*) blueberries [71].

Conversely, *Persicaria tinctoria* leaf powder exhibited greater long-term stability when stored at room temperature (25 °C) than samples subjected to hot air-drying methods. Similarly, natural colorant powders derived from red leaf amaranth (*Alternanthera amoena*) retained their color for a longer duration when stored at 28 °C, whereas higher temperatures of 45 °C or 55 °C led to faster degradation of the colorant [72].

In vitro cytotoxicity assays using cell lines are widely employed to evaluate the cytotoxic effects of natural compounds, chemicals, and potential drug candidates. These assays offer several advantages, including rapid results, cost-effectiveness, and avoidance of animal testing. One key parameter in cytotoxicity studies is the 50% cytotoxic concentration (CC_50_), which represents the concentration at which 50% of the tested cells are killed or inhibited. This metric is crucial in virology and pharmacology for assessing the toxicity and therapeutic potential of compounds.

The National Cancer Institute (NCI) classifies cytotoxic compounds based on their potency as follows: highly active (IC_50_ ≤ 20 μg/mL), moderately active (IC_50_ = 21–200 μg/mL), weakly active (IC_50_ = 201–500 μg/mL), and inactive (IC_50_ > 501 μg/mL) [73]. Based on this classification, the dye extracts from the 15 plant species in this study were grouped into three categories. Extracts from *Bixa orellana*, *Mangifera indica*, and *Strobilanthes cusia* were classified as inactive. Weakly active extracts included those from *Duabanga grandiflora*, *Oroxylum indicum*, *Phyllanthus emblica*, *Quercus brandisiana*, *Salix tetrasperma*, and *Terminalia chebula*. The final group, categorized as moderately cytotoxic, comprised the extracts from *Anneslea fragrans*, *Artocarpus lacucha*, *Biancaea sappan*, *Buchanania cochinchinensis*, *Curcuma longa*, and *Syzygium cumini*.

These findings align with previous research, which indicates that extracts from *Bixa orellana*, *Mangifera indica*, and *Strobilanthes cusia* do not exhibit genotoxicity [74]. Given their non-toxic nature, these three species hold promising potential for application in the food and pharmaceutical industries.

## 4. Materials and Methods

### 4.1. Study Sites

**Figure 7:**
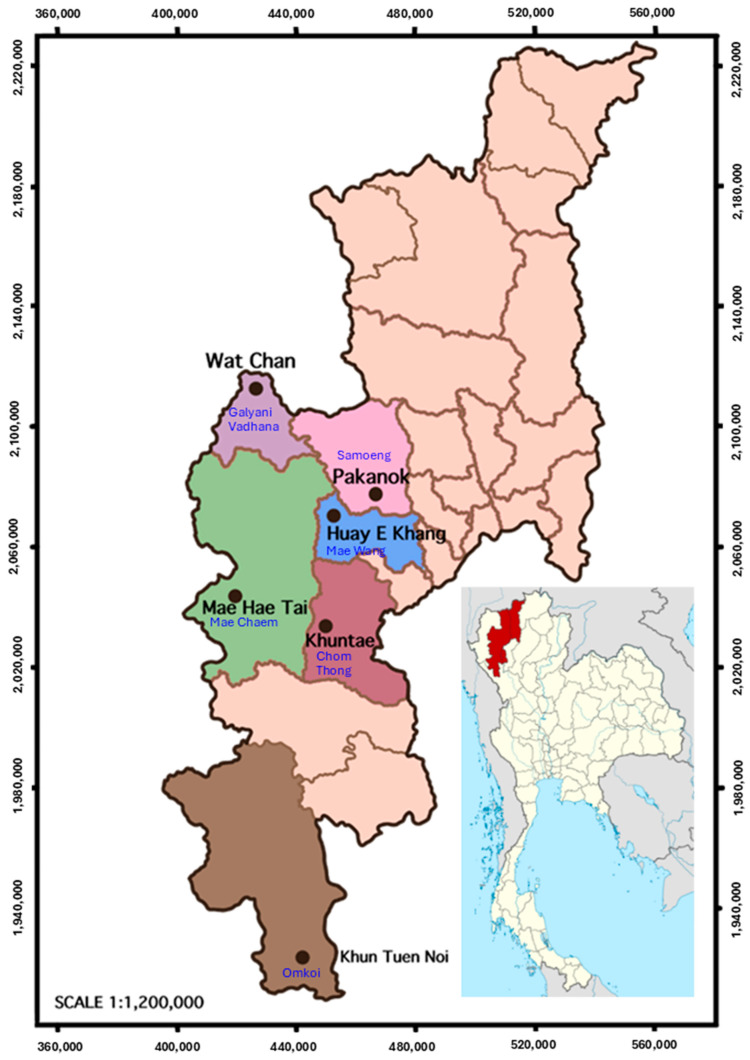
Location of study sites in Chiang Mai province in northern Thailand, where ethnobotanical field surveys on plant colorants were conducted in six Karen villages (black letters) in six different districts (blue letters).

**Figure 8:**
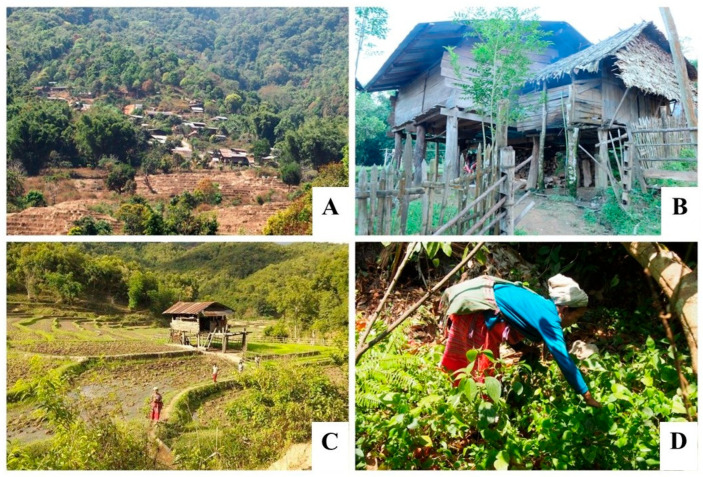
The Karen lifestyle and their livelihood. (**A**,**B**) Khun Tuen Noi village area and house; (**C**) cultivated rice fields in Huay E Khang village; (**D**) Karen women in Khuntae village forage vegetables in the forest.

The study was conducted in six Skaw Karen villages across six districts in Chiang Mai Province (Figure 7 and Figure 8 and Table 7). Most of our informants were farmers. These villages were selected based on three key criteria: they should be located in districts with a significant Karen population, the villagers should have an important reliance on forest resources for their livelihoods, and they should continue to practice and preserve traditional knowledge related to natural colorants. A preliminary survey was conducted to ensure that each village met these criteria before being included in the study.

### 4.2. The Ethnobotanical Study

Key informants with expertise in plant colorants, including local users, knowledgeable individuals, and traditional weavers, were selected using snowball sampling [75]. The selected informants ranged in age from 20 to 70 years, and their socioeconomic traits, such as gender, age, occupation, income, literacy, and formal education were documented. Semi-structured interviews were conducted to gather ethnobotanical data on plant colorants, including local plant names, plant parts used, preparation methods, color shades, and other applications.

Voucher specimens of the species yielding colorants were collected following standard procedures and deposited in the Queen Sirikit Botanic Garden (QBG) and Department of Biology, Chiang Mai University (CMUB) herbarium. Each plant was photographed, and the specimens were identified based on their morphological characteristics. The collected plant colorants were categorized according to their family, use category, plant parts used, and modes of preparation [76].

Ethnobotanical indices were calculated from the interview data to assess the significance of each plant colorant species. The use value (UV) was employed to determine the relative importance of each species [77]. Some plant species were found to produce multiple color shades, and the informant consensus on preferred shades was evaluated using the fidelity level (FL), which quantifies the agreement among informants regarding the most frequently used color shades. A higher FL indicates a greater consensus on a particular shade. Additionally, the Informant Consensus Factor (ICF), originally introduced by Trotten and Logan [78] and later refined by Heinrich et al. [79], was used to assess the homogeneity of knowledge among informants regarding the use of plant colorants for different color shades. This study was approved by the Chiang Mai University Research Ethics Committee, Thailand (certificate of approval number COA No. 026/64.

### 4.3. Colorants Extraction

Fifteen plant colorant species with the highest ethnobotanical index scores were selected for further analysis. All samples were dried in a hot air oven at 50 °C and then ground into a fine powder. The extraction process was carried out using the decoction method, in which 10 g of plant material was mixed with 100 mL of distilled water and boiled for one hour. The hot extract solution was then filtered through a fine cotton fabric to remove solid residues. The filtered dye solution was concentrated by evaporating the excess water in a pan at 80 °C. The concentrated solution was transferred to glass Petri dishes and further dried using a water bath with a temperature regulator set at 70 °C until a solid dye residue was obtained. The dried dye was then finely ground using a mortar and pestle [80,81]. The resulting solid dye powder was subsequently analyzed for cytotoxicity and color stability.

### 4.4. Color Stability Test

The solid dye powder was stored in amber glass vials under two temperature conditions: 25 °C and 45 °C. Measurements were recorded at 0, 15, and 30 days to assess color stability. Color changes were measured in triplicate using a MiniScan XE Plus spectrocolorimeter (HunterLab, Reston, VA, USA) [11]. The color difference (ΔE*) was calculated using the following equation:

$$∆E*={\left[{\left(∆L^{*}\right)}^{2}+{\left(∆a^{*}\right)}^{2}+{\left(∆b^{*}\right)}^{2}\right]}^{\frac{1}{2}}$$

where ΔE* represents the CIELAB color difference between the sample and the standard. ΔL* indicates the difference in lightness, with values ranging from 0 (black) to 100 (white), Δa* represents the shift between green (−a*) and red (+a*), and Δb* denotes the shift between yellow (+b*) and blue (−b*).

A ΔE* value of 0 signifies that the two colors are identical or indistinguishable to the human eye, while higher ΔE* values indicate increasing perceptible color differences.

### 4.5. In Vitro Cytotoxicity

The effects of plant colorant extract on cell viability were evaluated in vitro using the human hepatocarcinoma cell line HepG2 (ATCC^®^ HB-8065™) (ATCC, Manassas, VA, USA) as the model system. HepG2 cells were cultured in a growth medium and incubated at 37 °C in 5% CO₂. The dye powder was dissolved in DMSO, and plant extracts were prepared from a stock solution of 100 mg/mL in Eppendorf tubes. Cells were seeded in 96-well plates at a density of 2.5 × 10⁴ cells per well and incubated under the same conditions for 24 h.

After incubation, the cells were treated with the dye extract at final concentrations ranging from 1.6–1000 µg/mL and further incubated for 48 h (Jensen, 2009). Cell toxicity was assessed using PrestoBlue™ Cell Viability Reagents (Thermo Fisher Scientific, Waltham, MA, USA). Optical density (OD) was measured spectrophotometrically using an automatic plate reader at a test wavelength of 570 nm and a reference wavelength of 595 nm. The experiment included a negative control (cell culture medium) and a positive control (5% DMSO).

Cell viability was calculated relative to that of the untreated control, which was set at 100% viability. The half-maximal cytotoxic concentration (CC_50_) was determined using non-linear regression analysis with GraphPad Prism software v10.1.1 (USA) to evaluate the cytotoxicity of the dye powders.

## 5. Conclusions

While the Karen people still retain traditional knowledge of weaving for personal use, this expertise has declined significantly. Additionally, Karen people prefer pre-dyed yarn over naturally dyed yarn may contribute to the potential loss of this knowledge. The results of the color stability test and cytotoxicity data can be utilized to advise Karen weavers on enhancing their dyeing methods, thereby promoting their products effectively. Although several ethnobotanical studies have been conducted, they have primarily focused on medicinal plants or other specific plant-use categories. Although the diversity and importance of plant colorants used by various ethnic minorities in northern Thailand are well known, there is a lack of comprehensive studies that compile and compare the indigenous knowledge of these ethnic groups regarding plant colorants. Ethnobotanical studies focusing on traditional plant colorants among various ethnic groups in Thailand can provide valuable insights into the diversity and richness of plant colorants, enhance our understanding of traditional knowledge systems, and aid in preserving and promoting indigenous cultural heritage.

The development of natural colorants for food applications is a promising area of research. The selection of suitable plant-based colorants requires careful evaluation of factors such as safety, stability, availability, and environmental impact [8]. In this study, we used color stability and cytotoxicity tests to identify plant colorants with potential for food coloring development. Our goal was to identify plant species that not only exhibit stable color properties but also meet safety criteria, making them strong candidates for further research.

Among the tested species, *Oroxylum indicum* exhibited exceptional color stability, with minimal color change during storage. Its ΔE* values, when stored at both room temperature and 45 °C, were the lowest among all tested plant colorants, indicating superior stability. Regarding safety, O. indicum showed moderate cytotoxicity. However, beyond its use as a yellow natural colorant, its flowers and fruit are commonly consumed as food, while its bark is traditionally used for treating sore throats. Given its strong color stability and potential applications, this species presents a promising opportunity for further exploration and development as a natural food colorant across various industries.

## Figures and Tables

**Table 1:** Alphabetic list of plant colorant species used by the Karen in six villages in Chiang Mai province, Thailand. Voucher specimens were collected by Sukhumaabhorn Kaewsangsai (K.S.) and deposited at the QBG and CMUB herbaria.

Species Name (Voucher)	Family	Use Value (UV)	Life form	Village *	Part Use	Color	Mode of Preparation and Dye Extraction
*Adinandra integerrima* T.Anderson ex Dyer (K.S. 398)	Pentaphylacaceae	0.01	shrub	KT	fruit	pink	Pounded/Boiled in water
*Aeginetia indica* L. (K.S. 379)	Orobanchaceae	0.16	herb	KTN	flowers	purple, black, brown, grey	Pounded/Boiled in water
*Ageratina adenophora* (Spreng.) R.M.King & H.Rob. (K.S. 359)	Asteraceae	0.24	herb	KTN	whole plant	yellow, green	Pounded/Boiled in water
					bark	pink	peeling stem bark/Boiled in water
					leaves	green, yellow	Pounded/Boiled in water
*Albizia chinensis* (Osbeck) Merr. (K.S. 367)	Fabaceae	0.14	tree	HEK, KT, KTN	bark	yellow, brown, purple, red	Chopped/Boiled in water
*Anneslea fragrans* Wall. (K.S. 351)	Pentaphylacaceae	0.78	tree	HEK, KT, KTN, MHT, PKN, WC	bark	yellow, brown, red, orange, pink	Chopped/Boiled in water
*Artocarpus heterophyllus* Lam. (K.S. 368)	Moraceae	0.04	tree	HEK, WC	bark, stem	brown	Chopped/Boiled in water
*Artocarpus lacucha* Buch.-Ham. (K.S. 380)	Moraceae	0.38	tree	HEK, KT, MHT, PKN, WC	bark	brown, orange, pink, purple, red	Chopped/Boiled in water
*Baccaurea ramiflora* Lour. (K.S. 382)	Phyllanthaceae	0.02	shrub	HEK	bark	yellow, purple	Chopped/Boiled in water
*Biancaea sappan* (L.) Tod. (K.S. 369)	Fabaceae	0.57	tree	HEK, KT, MHT, PKN, WC	stem	orange, pink, purple, red, yellow	Chopped/Boiled in water
					bark	red	Chopped/Boiled in water
					fruit	brown	Pounded/Boiled in water
*Bixa orellana* L. (K.S. 352)	Bixaceae	0.36	shrub	HEK, KTN, PKN, WC	seed	yellow, orange, red	Pounded/Boiled in water
*Buchanania cochinchinensis* (Lour.) M.R.Almeida (K.S. 370)	Anacardiaceae	0.41	tree	HEK, PKN, WC	bark	red, brown, orange	Chopped/Boiled in water
					bark	orange	Chopped/Boiled in water
*Callicarpa arborea* Roxb. (K.S. 353)	Lamiaceae	0.09	tree	KTN, MHT	bark	yellow, brown, orange, purple, red	Chopped/Boiled in water
*Castanopsis acuminatissima* (Blume) A. DC. (K.S. 381)	Fagaceaae	0.01	tree	HEK	bark	brown	Chopped/Boiled in water
*Clerodendrum glandulosum* Lindl. (K.S. 354)	Lamiaceae	0.86	shrub	HEK, KT, KTN, MHT, PKN, WC	fruit	purple, blue, green	Pounded/Boiled in water
					leaves	green	Pounded/Boiled in water
*Curcuma longa* L. (K.S. 355)	Zingiberaceae	0.82	herb	HEK, KT, KTN, MHT, PKN, WC	rhizome	yellow, orange, green	Chopped/Boiled in water
*Dalbergia stipulacea* Roxb. (K.S. 397)	Fabaceae	0.05	scandent	HEK, KT, MHT	bark	brown, pink	Chopped/Boiled in water
*Dillenia obovata* (Blume) Hoogland (K.S. 402)	Dilleniaceae	0.11	tree	HEK, KT, WC	bark	red, brown, orange	Chopped/Boiled in water
*Diospyros mollis* Griff. (K.S. 383)	Ebenaceae	0.01	tree	HEK	fruit	black	Pounded/Boiled in water
*Dipterocarpus obtusifolius* Teijsm. ex Miq. (K.S. 371)	Dipterocarpaceae	0.01	tree	KTN	leaves	red	Boiled in water
*Duabanga grandiflora* Walp. (K.S. 366)	Lythraceae	0.40	tree	HEK, KTN, PKN, WC	bark	yellow, brown, green, grey, purple	Chopped/Boiled in water
*Gluta usitata* (Wall.) Ding Hou (K.S. 384)	Anacardiaceae	0.09	tree	HEK, WC	bark	brown	Chopped/Boiled in water
*Gmelina arborea* Roxb. (K.S. 372)	Lamiaceae	0.04	tree	HEK, KT, MHT	bark	green, yellow	Chopped/Boiled in water
*Harrisonia perforata* (Blanco) Merr. (K.S. 385)	Rutaceae	0.02	scandent	HEK, PKN	fruit	black	Pounded/Boiled in water
*Helicia nilagirica Bedd.* (K.S. 390)	Proteaceae	0.05	tree	MHT	bark	orange, brown	Chopped/Boiled in water
*Hibiscus sabdariffa* L. (K.S. 388)	Malvaceae	0.13	herb	HEK, KT, MHT, WC	calyx	red, pink	Boiled in water
*Hunteria zeylanica* Gardner ex Thwaites (K.S. 386)	Apocynaceae	0.04	shrub	KTN	leaves	green	Pounded/Boiled in water
*Indigofera tinctoria* L. (K.S. 373)	Fabaceae	0.07	shrub	MHT	leaves	blue	Pounded/Fermented in water
*Jatropha gossypifolia* L. (K.S. 389)	Euphorbiaceae	0.01	shrub	HEK	leaves	red	Pounded
*Lablab purpureus* (L.) Sweet (K.S. 357)	Fabaceae	0.23	herb	HEK, KT, KTN, WC	leaves	green	Pounded/Boiled in water
*Leucaena leucocephala* (Lam.) de Wit (K.S. 356)	Fabaceae	0.11	shrub	KTN	leaves	yellow, green	Boiled in water
*Livistona speciosa* Kurz	Arecaceae	0.21	palm	HEK, WC	fruit	grey	Pounded/Fermented in water
*Mangifera indica* L. (K.S. 358)	Anacardiaceae	0.53	tree	HEK, PKN, WC	bark	yellow, brown, green	Chopped/Boiled in water
*Melastoma malabathricum* L. (K.S. 360)	Melastomataceae	0.05	shrub	HEK, KTN, WC	fruit	purple, blue	Pounded/Fermented in water
*Morinda angustifolia* Roxb. (K.S. 365)	Rubiaceae			HEK, KT, KTN, MHT, WC	bark	red	Chopped/Boiled in water
					leaves	brown	Boiled in water
		0.63	shrub		root	brown, red, purple,	Chopped/Boiled in water
					stem	brown	Chopped/Boiled in water
*Morus alba* L. (K.S. 374)	Moraceae	0.39	shrub	HEK, KT, MHT, WC	fruit	purple, pink, grey	Pounded/Boiled in water
*Mucuna pruriens* (L.) DC. (K.S. 387)	Fabaceae	0.03	herb	HEK, MHT	leaves	grey	Pounded/Boiled in water
					stem	blue	Chopped/Boiled in water
*Musa × paradisiaca* L.	Musaceae	0.07	herb	HEK, KT, KTN	stem	purple, brown, grey	Chopped/Boiled in water
					fruit	grey	Chopped/Boiled in water
					leaves	grey	Chopped/Boiled in water
*Musa acuminata* Colla	Musaceae	0.04	herb	KTN, MHT	stem	grey	Chopped/Boiled in water
					leaves	black, grey	Chopped/Boiled in water
*Myrsine seguinii* H.Lév. (K.S. 375)	Primulaceae	0.07	shrub	HEK, WC	bark	brown	Chopped/Boiled in water
*Oroxylum indicum* (L.) Benth. ex Kurz (K.S. 361)	Bignoniaceae	0.73	tree	HEK, KT, KTN, MHT, PKN, WC	leaves	yellow	Pounded/Boiled in water
					bark	green, yellow	Chopped/Boiled in water
*Phyllanthus emblica* L. (K.S. 364)	Phyllanthaceae	0.44	shrub	HEK, KTN, MHT, PKN, WC	fruit	yellow, green, grey	Pounded/Boiled in water
					bark	grey	Chopped/Boiled in water
*Pterocarpus macrocarpus* Kurz (K.S. 401)	Fabaceae	0.01	tree	HEK	bark	purple	Chopped/Boiled in water
*Quercus brandisiana* Kurz (K.S. 376)	Fagaceaae	0.50	tree	HEK, KT, KTN, PKN, WC	bark	red, brown, orange, purple	Chopped/Boiled in water
*Quercus gomeziana* A.Camus (K.S. 392)	Fagaceaae	0.03	tree	KTN, MHT	bark	red, purple	Chopped/Boiled in water
*Salix tetrasperma* Roxb. (K.S. 393)	Salicaceae	0.22	tree	KTN	bark	red, brown, orange	Chopped/Boiled in water
*Saraca thailandica* Pongamornkul, Panyadee & Inta	Fabaceae	0.03	tree	KTN	bark	red, grey	Chopped/Boiled in water
					fruit	red	Pounded/Boiled in water
*Spondias pinnata* (L.f.) Kurz (K.S. 362)	Anacardiaceae	0.28	tree	HEK, WC	bark	grey	Chopped/Boiled in water
					fruit	brown, grey	Pounded/Boiled in water
*Strobilanthes cusia* Kuntze (K.S. 377)	Acanthaceae	0.93	herb	HEK, KT, KTN, MHT, PKN, WC	leaves	blue, green, grey	Pounded/Fermented in water
					root	green	Chopped/Boiled in water
*Syzygium cumini* (L.) Skeels (K.S. 363)	Myrtaceae	0.33	tree	HEK, MHT, PKN, WC	bark	red, grey, purple	Chopped/Boiled in water
					fruit	grey	Pounded/Boiled in water
*Terminalia chebula* Retz. (K.S. 394)	Combretaceae	0.33	tree	HEK, MHT, WC	fruit	grey, brown, green	Pounded/Boiled in water
					bark	grey	Chopped/Boiled in water
*Terminalia phillyreifolia* (Van Heurck & Müll.Arg.) Gere & Boatwr. (K.S. 400)	Combretaceae	0.01	tree	KTN	bark	brown	Chopped/Boiled in water
*Ternstroemia gymnanthera* (Wight & Arn.) Sprague (K.S. 378)	Pentaphylacaceae	0.03	shrub	KTN	bark	red, purple	Chopped/Boiled in water

* Village: KT = Khuntae; KTN = Khun Tuen Noi; HEK = Huay E Khang; MHT = Mae Hae Tai; PKN = Pakanok and WC = Wat Chan.

**Table 2:** Color shades and fidelity levels (FL) for each of 52 colorant species used by the Karen in six villages in Chiang Mai in northern Thailand.

Species Name	Fidelity Levels (FL) of Each Color Shade (%)										
	Black	Blue	Brown	Green	Grey	Orange	Pink	Purple	Red	Yellow	Use Report
*Adinandra integerrima*	-	-	-	-	-	-	100	-	-	-	1
*Aeginetia indica*	6	-	6	-	6	-	-	82	-	-	18
*Ageratina adenophora*	-	-	-	39	-	-	4	-	-	57	28
*Albizia chinensis*	-	-	75	-	-	-	-	13	6	6	16
*Anneslea fragrans*	-	-	48	-	-	15	8	-	27	2	90
*Artocarpus heterophyllus*	-	-	100	-	-	-	-	-	-	-	5
*Artocarpus lacucha*	-	-	41	-	-	2	9	2	46	-	44
*Baccaurea ramiflora*	-	-	-	-	-	-	-	50	-	50	2
*Biancaea sappan*	-	-	2	-	-	3	85	2	5	3	65
*Bixa orellana*	-	-	-	-	-	88	-	-	2	10	41
*Buchanania cochinchinensis*	-	-	74	-	-	17	-	-	9	-	47
*Callicarpa arborea*	-	-	10	-	-	30	-	30	20	10	10
*Castanopsis acuminatissima*	-	-	100	-	-	-	-	-	-	-	1
*Clerodendrum glandulosum*	-	63	-	35	-	1	-	1	-	-	99
*Curcuma longa*	-	-	-	1	-	6	-	-	-	93	94
*Dalbergia stipulacea*	-	-	67	-	-	-	33	-	-	-	6
*Dillenia obovata*	-	-	62	-	-	31	-	-	7	-	13
*Diospyros mollis*	100	-	-	-	-	-	-	-	-	-	1
*Dipterocarpus obtusifolius*	-	-	-	-	-	-	-	-	100	-	1
*Duabanga grandiflora*	-	-	4	2	68	-	-	24	-	2	46
*Gluta usitata*	-	-	100	-	-	-	-	-	-	-	10
*Gmelina arborea*	-	-	-	40	-	-	-	-	-	60	5
*Harrisonia perforata*	100	-	-	-	-	-	-	-	-	-	2
*Helicia nilagirica*	-	-	33	-	-	67	-	-	-	-	6
*Hibiscus sabdariffa*	-	-	-	-	-	-	73	-	27	-	15
*Hunteria zeylanica*	-	-	-	100	-	-	-	-	-	-	5
*Indigofera tinctoria*	-	100	-	-	-	-	-	-	-	-	8
*Jatropha gossypifolia*	-	-	-	-	-	-	-	-	100	-	1
*Lablab purpureus*	-	-	-	100	-	-	-	-	-	-	27
*Leucaena leucocephala*	-	-	-	46	-	-	-	-	-	54	13
*Livistona speciosa*	-	-	-	-	100	-	-	-	-	-	24
*Mangifera indica*	-	-	53	13	-	-	-	-	-	34	61
*Melastoma malabathricum*	-	50	-	-	-	-	-	50	-	-	6
*Morinda angustifolia*	-	-	24	-	-	15	-	3	58	-	72
*Morus alba*	-	-	-	-	58	-	22	20	-	-	45
*Mucuna pruriens*	-	33	-	-	67	-	-	-	-	-	3
*Musa × paradisiaca*	-	-	13	-	62	-	-	25	-	-	8
*Musa acuminata*	40	-	-	-	60	-	-	-	-	-	5
*Myrsine seguinii*	-	-	100	-	-	-	-	-	-	-	8
*Oroxylum indicum*	-	-	-	75	-	-	-	-	-	25	84
*Phyllanthus emblica*	-	-	-	8	88	-	-	-	-	4	51
*Pterocarpus macrocarpus*	-	-	-	-	-	-	-	100	-	-	1
*Quercus brandisiana*	-	-	79	-	-	14	-	5	2	-	58
*Quercus gomeziana*	-	-	-	-	-	-	-	25	75	-	4
*Salix tetrasperma*	-	-	28	-	-	16	-	-	56	-	25
*Saraca thailandica*	-	-	-	-	33	-	-	-	67	-	3
*Spondias pinnata*	-	-	12	-	88	-	-	-	-	-	32
*Strobilanthes cusia*	-	70	-	28	2	-	-	-	-	-	107
*Syzygium cumini*	-	-	-	-	77	-	-	18	5	-	38
*Terminalia chebula*	-	-	11	5	84	-	-	-	-	-	38
*Terminalia phillyreifolia*	-	-	100	-	-	-	-	-	-	-	1
*Ternstroemia gymnanthera*	-	-	-	-	-	-	-	50	50	-	4

**Table 3:** Informant consensus factor (ICF) value for dyes recorded in six Karen villages in Chiang Mai, Thailand.

Colors/Shades	No. of Use Reports	No. of Species	ICF
blue	149	6	0.97
grey	229	14	0.94
green	195	14	0.93
yellow	169	15	0.92
brown	263	24	0.91
pink	92	9	0.91
orange	102	14	0.87
red	128	19	0.86
purple	65	18	0.73
black	6	5	0.20

**Table 4:** List of plant colorant species used by the Karen in other applications apart from dyeing.

Species	Use Category	Part Used	Application
*Ageratina adenophora*	medicine	leaves	crushed and applied to wounds to stop bleeding
*Aeginetia indica*	food	flowers	mixed with sticky rice to make a dessert
*Albizia chinensis*	material	bark	crushed and soaked in water to use as shampoo
*Anneslea fragrans*	social use	leaves	chewed with betel nut
*Artocarpus heterophyllus*	food	fruit	eaten as fruit
*Artocarpus lacucha*	social use	bark	chewed with betel nut
	food	fruit	eaten as fruit
*Baccaurea ramiflora*	food	fruit	eaten as fruit
*Biancaea sappan*	medicine	stem	decocted and drunk as a blood tonic and nourishing remedy for the body
*Buchanania cochinchinensis*	food	fruit	eaten as fruit
*Castanopsis acuminatissima*	material	stem	construction
*Clerodendrum glandulosum*	food	young shoot	cooked and eaten with chili paste
*Curcuma longa*	food	rhizome	added to food
	medicine	rhizome	ground into powder and eaten to treat gastric
*Dipterocarpus obtusifolius*	medicine	exudate	eaten to treat sore throat
*Duabanga grandiflora*	material	stem	construction
*Gmelina arborea*	food	flowers	mixed with sticky rice to make a dessert
*Helicia nilagirica*	medicine	leaves	decocted and used to wash eye
*Hibiscus sabdariffa*	food	calyx	boiled and drunk as beverage
*Lablab purpureus*	material	stem	mashed to make a rope
	food	fruit	cooked and eaten with chili paste
*Leucaena leucocephala*	food	fruit	eaten as fruit
*Livistona speciosa*	material	leaves	roof construction
*Melastoma malabathricum*	food	fruit	eaten as fruit
*Morinda angustifolia*	medicine	root	decocted and drunk as a tonic and detox
*Morus alba*	food	leaves	boiled and drunk as tea
		fruit	eaten as fruit
*Musa × paradisiaca*	food	fruit	eaten as fruit
*Oroxylum indicum*	food	young fruit, flowers	cooked and eaten with chili paste
	medicine	fruit	eaten to treat sore throat
*Phyllanthus emblica*	medicine	fruit	eaten raw to treat sore throat
*Pterocarpus macrocarpus*	material	stem	construction
*Quercus brandisiana*	material	resin	lacquered wood
*Saraca thailandica*	food	fruit	cooked and eaten with chili paste
*Spondias pinnata*	food	young shoot	eaten raw with chili paste
		fruit	added to food
*Syzygium cumini*	food	fruit	eaten as fruit
*Terminalia chebula*	medicine	fruit	eaten raw to treat splenomegaly

**Table 5:** Effect of 120 days storage time and temperature on the ΔE* values.

Species Name	Control	45 °C
*Anneslea fragrans*	5.78	6.38
*Artocarpus lacucha*	5.87	6.53
*Biancaea sappan*	6.74	5.86
*Bixa orellana*	7.13	5.68
*Buchanania cochinchinensis*	6.54	5.74
*Curcuma longa*	6.62	5.35
*Duabanga grandiflora*	5.90	5.42
*Mangifera indica*	6.52	6.21
*Oroxylum indicum*	4.92	4.64
*Phyllanthus emblica*	8.86	7.29
*Quercus brandisiana*	5.50	5.87
*Salix tetrasperma*	5.50	5.67
*Strobilanthes cusia*	7.54	5.93
*Syzygium cumini*	5.88	6.01
*Terminalia chebula*	5.46	5.84
Average	6.32 ± 0.96	5.90 ± 0.57

**Table 6:** Cytotoxicity (CC_50_) of the 15 different plant species extracted.

Species Name	Family	CC_50_ (µg/mL)
*Artocarpus lacucha*	Moraceae	63.96
*Buchanania cochinchinensis*	Anacardiaceae	70.71
*Biancaea sappan*	Fabaceae	76.73
*Curcuma longa*	Zingiberaceae	118.2
*Anneslea fragrans*	Pentaphylacaceae	137.0
*Syzygium cumini*	Myrtaceae	185.4
*Salix tetrasperma*	Salicaceae	207.6
*Quercus brandisiana*	Fagaceaae	211.2
*Duabanga grandiflora*	Lythraceae	223.0
*Phyllanthus emblica*	Phyllanthaceae	263.6
*Terminalia chebula*	Myrtaceae	278.4
*Oroxylum indicum*	Bignoniaceae	294.4
*Mangifera indica*	Anacardiaceae	634.5
*Bixa orellana*	Bixaceae	659.1
*Strobilanthes cusia*	Acanthaceae	994.1

**Table 7:** Basic information of six Karen villages in Chiang Mai province where the ethnobotanical study was conducted.

Village	Khun Tuen Noi	Khuntae	Mae Hae Tai	Huay E Khang	Wat Chan	Pakanok
District	Omkoi	Chom Thong	Mae Chaem,	Mae Wang	Galyani Vadhana	Samoeng
Coordinates	17.315556 N 98.332633 E	18.391561 N 98.506530 E	18.429267 N 98.138613 E	18.725907 N 98.568085 E	19.074644 N 98.304557 E	18.514873 N 98.275978 E
Altitude (MASL)	1441	1228	1112	922	977	863
Household #	37	229	201	111	172	67
Population #	182	807	937	525	633	278
Distance from nearest urban center (km)	109	26	45	35	6.6	17

## Data Availability

Data is contained within the article.

## Supplementary Materials

The following supporting information can be downloaded at: https://www.mdpi.com/article/10.3390/plants14091348/s1, Figure S1: The cytotoxicity of 15 different plant extracts on HepG2 cells.
