# Supplementary material for: Ethnobotany, Cytotoxicity and Color Stability of Karen Natural Colorants
Source: Plants (Basel). 2025 Apr 29;14(9):1348. doi: 10.3390/plants14091348 (PMC12073458; doi:10.3390/plants14091348)
Supplement: Supplementary file 1 [file plants-14-01348-s001.zip › plants-3568944-supplementary.pdf]

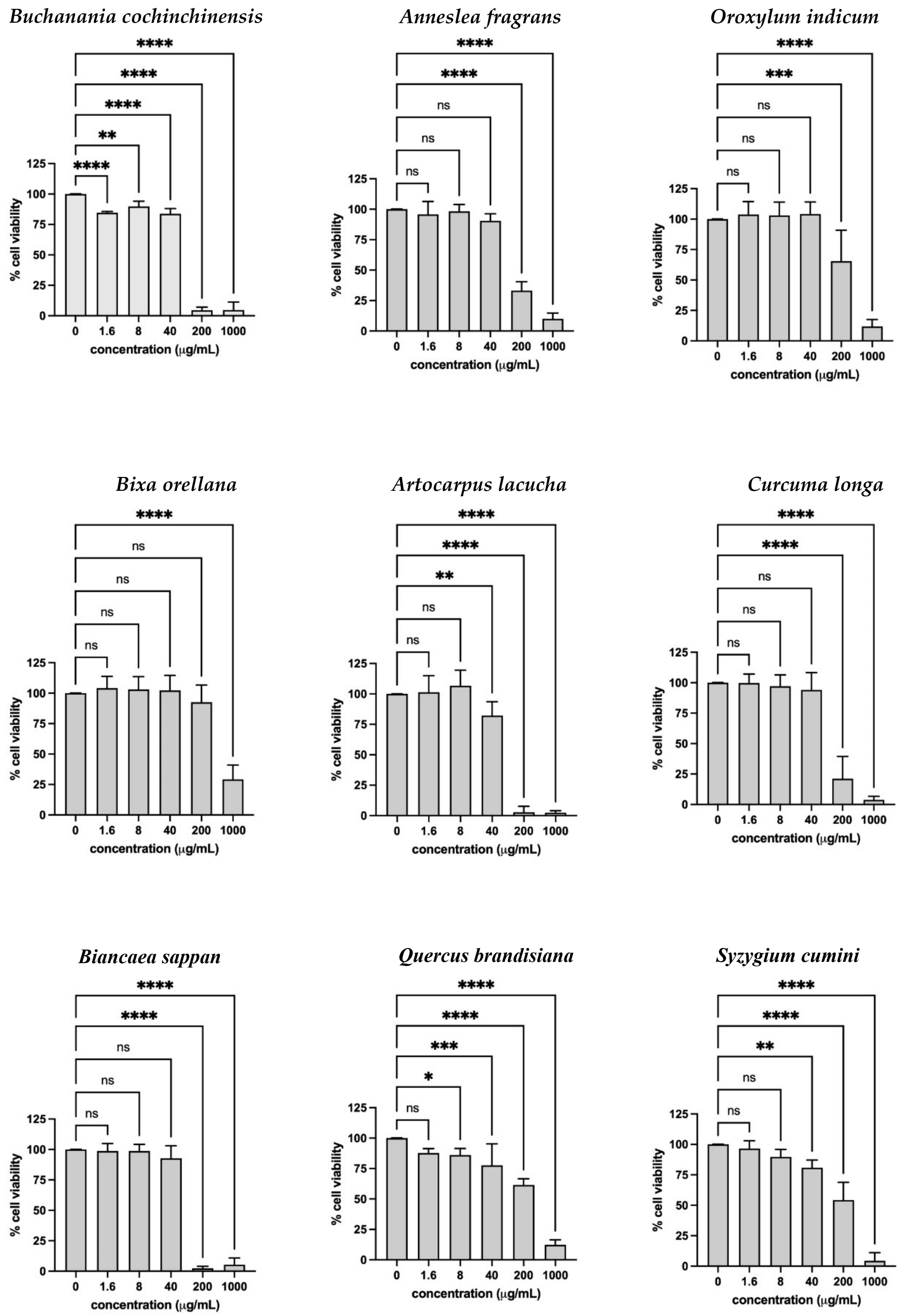

Supplementary Figure S1. The cytotoxicity of 15 different plant extracts on HepG2 cells. HepG2 cells were treated with plant extracts at the concentrations varied from 0-1000 μg/mL. The cell viability was measured at 48 hours after incubation and analyzed for % cell viability relative to non-treatment control (set as 100%). Experiments were performed independently three times (N = 3). Statistical significance is indicated as *p < 0.05, **p < 0.01, *** p < 0.001, and ****p < 0.0001.

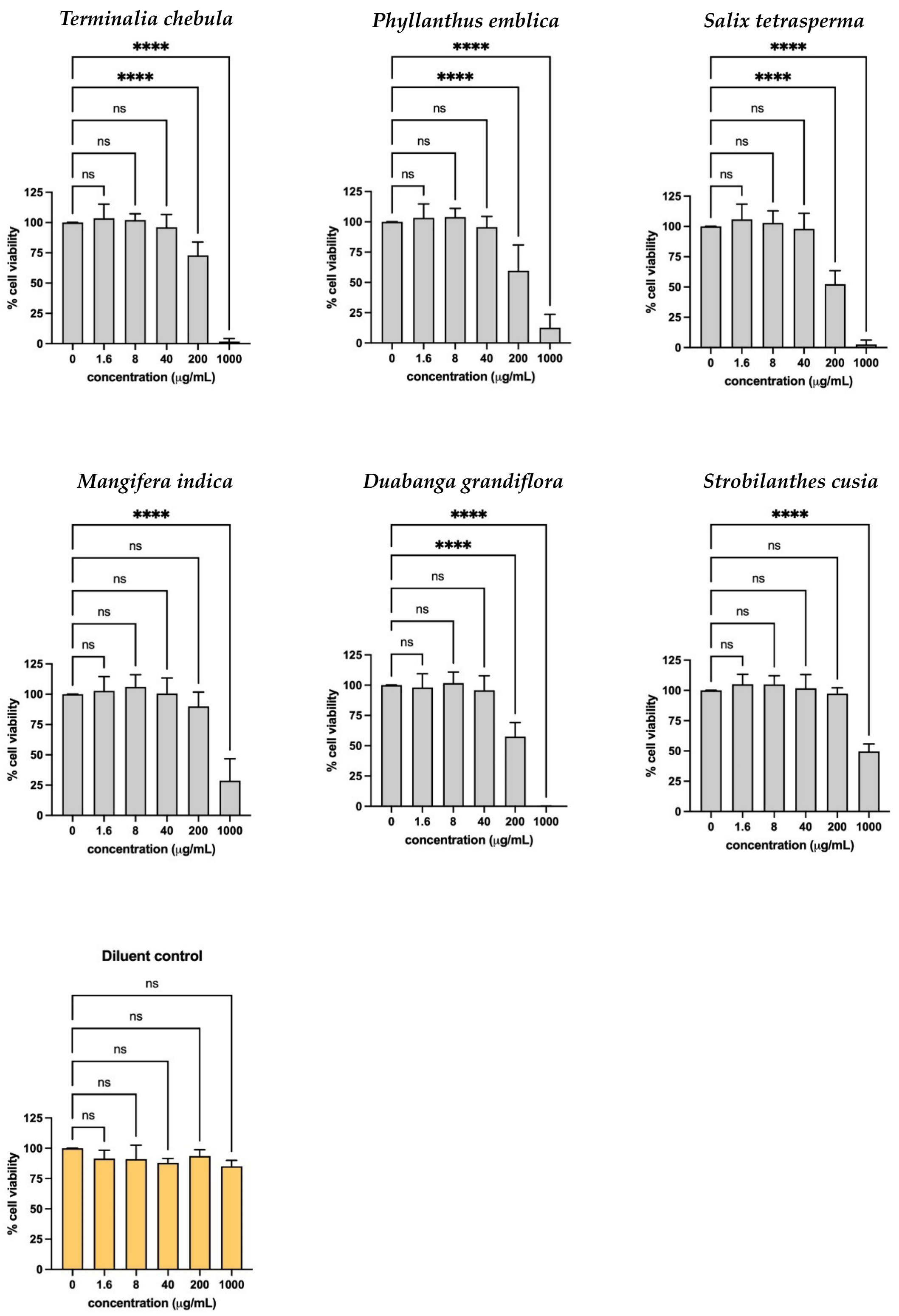

Supplementary Figure S1 (cont.). The cytotoxicity of 15 different plant extracts on HepG2 cells. HepG2 cells were treated with plant extracts at the concentrations varied from 0-1000 μg/mL. The cell viability was measured at 48 hours after incubation and analyzed for % cell viability relative to non-treatment control (set as 100%). Experiments were performed independently three times (N = 3). Statistical significance is indicated as *$p < 0.05$, **$p < 0.01$, *** $p < 0.001$, and ****$p < 0.0001$.
